# Characteristics of Adults with Down Syndrome: Prevalence of Age-Related Conditions

**DOI:** 10.3389/fmed.2014.00051

**Published:** 2014-12-03

**Authors:** Angelo Carfì, Manuela Antocicco, Vincenzo Brandi, Camilla Cipriani, Francesca Fiore, Donatella Mascia, Silvana Settanni, Davide L. Vetrano, Roberto Bernabei, Graziano Onder

**Affiliations:** ^1^Department of Geriatrics, Centro Medicina dell’Invecchiamento, Università Cattolica del Sacro Cuore, Rome, Italy

**Keywords:** Down syndrome, geriatric assessment, aging, premature, multimorbidity, geriatric syndromes

## Abstract

**Introduction:** In the last decades, life expectancy of persons with Down syndrome (DS) has dramatically increased and it is estimated that they will be living as long as the general population within a generation. Despite being included among the progeroid syndromes, because of the presence of features typically observed in older adults, DS is still regarded as a disease of pediatric interest. Because limited knowledge is available on the clinical characteristics of adults with DS, this study aimed to assess clinical and non-clinical features of this population and to describe similarities to the geriatric population.

**Methods:** In this study, we described 60 adults with DS evaluated at the Day Hospital of the Geriatric Department of the Policlinico A. Gemelli, Università Cattolica del Sacro Cuore in Rome. Individuals were assessed through a standardized protocol.

**Results:** The mean age of study participants was 38 years (range, 18–58 years) and 42 (70.0%) were women. Geriatric conditions were highly prevalent: severe cognitive impairment was diagnosed in 39 (65.0%) participants, behavioral symptoms were present in 25 (41.7%), and functional impairment in 23 (38.3%). Six (10.0%) participants lived in institutions and 11 (18.3%) were diagnosed as obese. The mean number of drugs used was 2.4; use of psychotropic drugs was highly prevalent. The most common chronic diseases were thyroid problems (44, 73.3%), followed by mood disorders (19, 31.7%), osteoporosis (18, 30.0%), and cardiac problems (10, 16.7%). Geriatric conditions and chronic diseases were more prevalent among participants aged ≥40 years.

**Conclusion:** Several similarities between older adults and adults with DS were observed. Comorbidities, geriatric conditions, cognitive and functional deficits, and social problems are highly prevalent in both populations, contributing to the high complexity of these patients’ assessment and treatment.

## Introduction

People with Down syndrome (DS) are known to have shortened life spans. In the last century, however, their life expectancy has dramatically increased, from 9 years in 1929 (1) to 60 years in 2002 (2–4). It was estimated that persons with DS will be living as long as the general population within a generation (5).

This improvement in survival has been attributed to three major changes in the approach to persons with DS. First, their vulnerability to respiratory infections, due to an impaired immune system, has been addressed by the widespread availability of antibiotic therapies since 1950s. Beginning in the 1970s, the prevention of respiratory infections was improved further by the trend toward deinstitutionalization; DS patients were moved into home care or sheltered housing. Congenital heart problems, which affect almost half of the persons with DS and are responsible for the high rate of early mortality, have been treated increasingly with early heart surgery since 1980s.

Given this demographic change, DS can no longer be considered a “pediatric” disease; rather it is a condition that affects the whole life span. So far, researchers have studied comprehensively pediatric conditions related to DS; however, it is now time to focus investigations on the characteristics and treatment of adults with this condition.

Adults with DS are known to age prematurely and to present with chronic conditions resembling those of their elders. A model of biological age vs. chronological age showed that people with DS age both earlier and faster than healthy controls (6). Indeed, DS is considered a segmental progeroid syndrome (7, 8), in which the premature aging process affects selected organ systems, including the central nervous, immune, respiratory, gastrointestinal, musculoskeletal, urinary, endocrine, vision and hearing systems (9). During their life span, persons with DS experience somatic degenerative changes such as hair graying and loss, increased tissue lipofuscin, variations in the distribution of adipose tissue, amyloidosis, increased autoimmunity, and cataracts; these changes occur earlier than in persons with other types of intellectual disabilities (10). Histological changes in the central nervous system (CNS) are almost identical to those seen in Alzheimer’s disease (AD) and appear prematurely in persons with DS.

Premature aging in DS produces patterns of comorbidities similar to those found in the elderly patient. Common conditions encountered in adults with DS include: AD, epilepsy, mood and behavioral disorders, visual and hearing impairment, osteoporosis, osteoarthritis, and autoimmune diseases, such as thyroiditis and celiac disease. Trajectories of disease, which compare DS with general populations, now show increased prevalence and premature onset of visual and hearing impairment, epilepsy, thyroid disorders, and dementia in the DS population (11).

Despite the fact that clinical characteristics of adults with DS seem to resemble those of the geriatric population, to date, limited research has systematically assessed the chronic diseases and geriatric conditions affecting this population. The present study aims to describe the clinical and non-clinical characteristics of adults with DS evaluated at the Day Hospital (DH) of the Geriatric Department of the Policlinico A. Gemelli, Università Cattolica del Sacro Cuore in Rome, focusing on geriatric conditions, including functional and cognitive deficits, and comorbidities.

## Materials and Methods

### Participants

Participants were adults with DS, aged 18 years or older, assessed at the DH of the Geriatric Department of the Policlinico A. Gemelli, Università Cattolica del Sacro Cuore in Rome. No specific inclusion criterion was required to be admitted to the DH except for age 18 years or older. Participants were referred to the DH by DS associations and family physicians.

Informed consent was obtained from all participants. The surrogate legal representative was asked to get the information and sign the consent form in those cases where individuals were cognitively impaired or unable to make the decision for themselves. The study was approved by the Ethical Committee of the Università Cattolica del Sacro Cuore.

All adults with DS received a comprehensive clinical assessment, following a standardized protocol that included physical exam, EKG, blood sampling, formal neuropsychological evaluation, echocardiography, DEXA scan, nutritional assessment and in selected cases hearing evaluation, pulmonary evaluation for obstructive sleep apnea syndrome, and gastroenterological evaluation for celiac disease.

Cognitive status was assessed using the Wechsler Adult Intelligence Scale [WAIS-IV (12)]. This scale was shown to be a valid and reliable instrument to assess cognitive status in persons with intellectual disabilities; a score <45 defined severe cognitive impairment. Functional status was evaluated using Basic Activities of Daily Living (ADL) (13). This scale was largely used to assess functional status in persons with intellectual disabilities (14–16). Functional impairment was defined as dependency in 1 or more ADLs. Nutritional status was assessed using body mass index; malnutrition defined as BMI <18.5 kg/m^2^ and obesity as BMI ≥30 kg/m^2^. Behavioral symptoms were considered to be present if the participant was using an antipsychotic drug or exhibited one or more symptoms in the last 3 days prior to the assessment: wandering, verbal abuse (i.e., threatening, screaming at or cursing others); physical abuse (i.e., hitting, shoving, scratching, or sexually abusing others); socially inappropriate or disruptive behavior (i.e., include making disruptive sounds or noises, screaming out, smearing or throwing food or feces, hoarding, and rummaging through others’ belongings); resisting care (i.e., include verbal or physical resistance to taking medications, taking injections, completing a variety of ADL, or eating).

Chronic conditions included: epilepsy, thyroid problems, osteoporosis, mood disorders, and cardiac problems (including history of congenital heart defects, history of heart surgery, heart failure, and atrial fibrillation). Chronic conditions were assessed by gathering information from the participant, the general practitioner, and by careful review of charts. Psychotropic drugs included antipsychotics, benzodiazepines, antiepileptics, and antidepressants.

Assessment was performed by trained clinical staff. Data from 60 adults with DS that completed the clinical assessment (see below) are presented in this manuscript.

### Data analysis

For comparisons, the study population was divided in these two age groups: ≥40 and <40 years of age. These groups were based on the increased prevalence of chronic diseases, including AD, after 40 years of age (17–19). To compare characteristics of participants based on age groups, we used ANOVA analyses for normally distributed variables, non-parametric Mann–Whitney *U*-test for skewed variables, and Fisher’s exact test for dichotomous variables. Data were analyzed using SPSS (version 18.0).

## Results

The mean age of the 60 adults with DS was 38 years (range 18–58 years) and 42 (70%) were women. In the <40 years group were 33 participants (mean age, 29.5 ± 6.4; 57.6% females), whereas there were 27 in the ≥40 years group (mean age, 46.0 ± 4.6; 85.2% females). The main characteristics of study population according to age group are presented in Table 1.

**Table 1:** **Characteristics of the study population according to age group**.

	Age <40 years; *n* = 33 (55%)	Age ≥40 years, *n* = 27 (45%)	*p*-value
Demographic characteristics
Age	29.5 ± 6.4	46.0 ± 4.6	<0.001
Female gender	19 (57.6%)	23 (85.2%)	0.020
Geriatric conditions
Severe cognitive impairment	26 (78.8%)	13 (48.1%)	0.001
Behavioral symptoms	11 (33.3%)	14 (51.9%)	0.191
Functional impairment	8 (24.2%)	15 (55.6%)	0.017
Number of impaired ADL	0.5 ± 1.1	1.5 ± 1.5	0.005
Institutionalization	0 (0.0%)	6 (22.2%)	0.006
Number of drugs used	2.0 ± 1.2	3.0 ± 1.4	0.016
Use of any psychotropic drugs	6 (18.2%)	17 (63.0%)	0.001
Nutritional problems
Malnutrition (BMI <18.5 kg/m^2^)	1 (3.0%)	0 (0.0%)	1.000
Obesity (BMI ≥30.0 kg/m^2^)	4 (12.1%)	7 (25.9%)	0.197
Chronic diseases
Epilepsy	1 (3.0%)	5 (18.5%)	0.081
Thyroid problems	24 (72.7%)	20 (74.1%)	1.000
Osteoporosis	7 (21.2%)	11 (40.7%)	0.156
Mood disorders	8 (24.2%)	11 (40.7%)	0.265
Cardiac problems	4 (12.1%)	6 (22.2%)	0.322

*Values are reported as mean ± SD or frequency (%)*.

**Figure 1:**
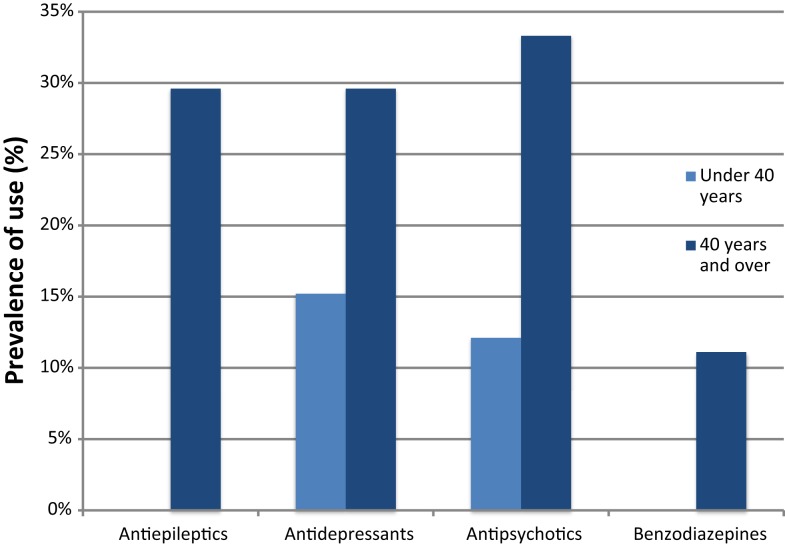
**Prevalence of use of psychotropic drugs by age groups**.

Geriatric conditions were common in both groups. Severe cognitive impairment was diagnosed in 26 (78.8%) participants in the <40-year age group and in 13 (48.1%) of the ≥40 group. Behavioral symptoms were present in 11 (33.3%) and 14 (51.9%) of the <40 and ≥40 groups, respectively. Eight (24.2%) persons were functionally impaired in the <40 group, in contrast with 15 (55.6%) in the ≥40 group. Six (22.2%) participants in the ≥40 group lived in institutions. Malnutrition was uncommon, whereas obesity was more prevalent, affecting 4 (12.1%) and 7 (25.9%) individuals in the <40 and ≥40 groups, respectively. The mean number of drugs used was 2.0 in the younger group and 3.0 in the older group, with a high prevalence of psychotropic drug use, particularly in those ≥40 years of age (Figure 1).

**Figure 2:**
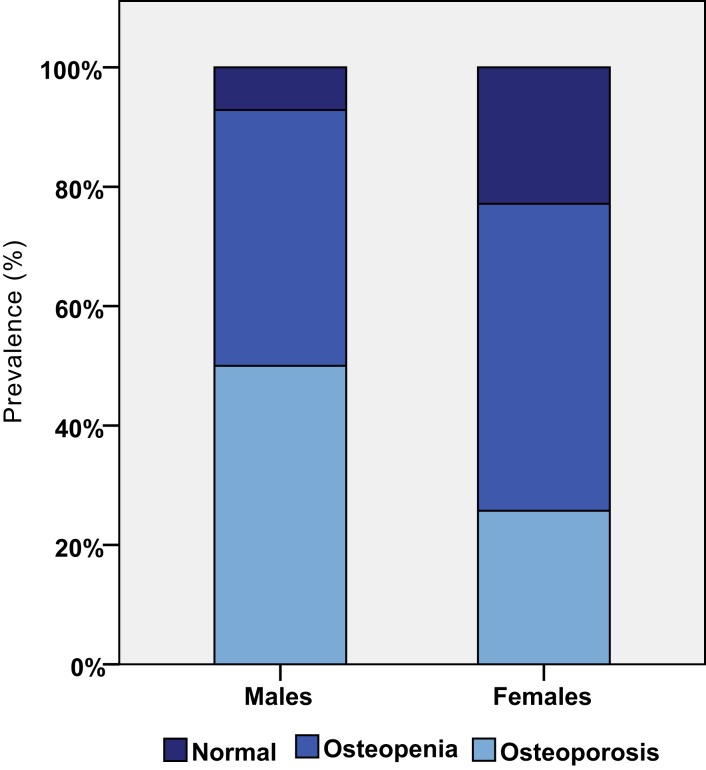
**Prevalence of osteopenia and osteoporosis by gender**.

The most common chronic diseases were thyroid problems, followed by mood disorders and osteoporosis. Figure 2 shows that osteoporosis was a common condition in both males and females. Epilepsy was more prevalent in the ≥40 group, affecting five (18.5%) persons compared with 1 (3%) of the <40 group. Finally, cardiac problems were diagnosed in four (12.1%) persons of the <40 group and in six (22.2%) of the ≥40 group. Interestingly, none of the participants was diagnosed with ischemic heart disease or stroke.

## Discussion

Down syndrome has been historically considered as a pediatric condition. This was due to the very short life span of this population in the past, with only a few people surpassing 18 years of age. As the life expectancy of this population increased, new health issues emerged, making the care of this population particularly challenging. Adults with DS are characterized by the presence of several concomitant, overlapping clinical conditions; they usually receive multiple medications and treatments, in particular, psychotropic medications, and sometimes face inadequate social and family support. The complexity of this population is further complicated by the presence of functional and cognitive impairments, which increase the risk of developing specific syndromes, including behavioral and nutritional problems. Prevalence of these conditions increases after 40 years of age. This complexity of care required by this patient population surpasses the traditional practice of medicine and it resembles care that is usually required by older adults (11).

Indeed, there is growing debate about which type of providers delivers the highest quality of care for adults with DS and a wide variety of comorbidities (11, 20). Families and associations often feel abandoned by institutions and health services as their relatives and assisted persons surpass the age of 18 years. Given the high prevalence of comorbidities, functional and cognitive impairment, polypharmacy, and geriatric conditions, geriatric care might be considered as a possible option for the care of adults with DS.

Geriatric care has been shown to have a relevant impact on the assessment and treatment of overlapping conditions and this effect is independent of patient age. Comprehensive Geriatric Assessment (CGA) can evaluate the diverse problems facing the adult with DS, including his/her comorbidities, syndromes, socio-economic problems, and functional and cognitive deficits, which are not usually addressed by traditional medical assessments. CGA provides a more individualized and comprehensive care plan for a single patient. CGA and geriatric care in adults with DS have the potential to increase diagnostic accuracy; optimize medical treatment; improve prognosis; restore, maintain, and maximize functional autonomy; compensate for the loss of autonomy with appropriate support services; improve quality of life; and ultimately reduce costs of care.

A model of care already adopted for complex older adults could thus be tested and implemented in adults with DS. Such a model could be based on a close interaction between general practitioners and a team of geriatricians trained in multidimensional assessment of adults with DS. While the general practitioner would ensure the continuity of care, the geriatric team would simplify the transition process from pediatric to adult care coordinating the multidisciplinary care for complex and chronic conditions.

Indeed, the care of adults with DS is still an open research field and researchers need to address several issues:

1. Clear data on the prevalence of chronic diseases and geriatric conditions (i.e., falls and fractures, behavioral symptoms, nutritional problems, etc.) are lacking, and large epidemiological studies are needed to identify risk factors in this population. Indeed, as observed for older adults, “traditional” risk factors, derived from a general population, might either be weakly associated with negative events or positively affect clinical outcomes.
2. Protocols for screening for chronic diseases and conditions are lacking. Standardized protocols are available for screening children with DS for most common diseases; however, a general consensus on which chronic diseases and conditions should be screened, available, and valid screening tools, appropriate age to start screening and frequency of screening are not available for adults with DS.
3. Little is known about treatment of diseases and conditions observed in adults with DS. For example, clinical trials showed that treatment with donepezil (21) or memantine (22) does not improve cognition in AD-like dementia (23–26). Osteoporosis is an extremely prevalent disease in adults with DS (27–32), but no data are available on its effective treatment. Indeed, low bone formation and decreased bone turnover, rather than increased bone resorption, could be the primary cause of osteoporosis in adults with DS (27). Therefore, it might be hypothesized that agents that inhibit bone resorption (i.e., bisphosphonates and denosumab) might not be the best choice in this population. This lack of data on effective treatment for common diseases from trials in the DS population, a further similarity to the geriatric population, will require physicians to generalize from findings obtained from a general population. However, the extrapolation of evidence from studies performed in a general population to subgroups of patients (i.e., complex older adults or adults with DS), is not straightforward and should consider multiple issues, including patients’ risk factors and competing diseases and conditions.

An important limitation of this study relates to generalizability of the results. Our findings, which are based on a sample of adults with DS referred for assessment to a single center, which cannot be representative of the whole population of adults with DS.

In conclusion, the present study suggests several similarities between complex older adults and adults with DS. Comorbidities, geriatric conditions, cognitive and functional deficits, and social problems are highly prevalent in both populations. Due to these similarities, geriatric care based on CGA may provide the most appropriate care to persons with DS as they grow to adulthood. Further research is needed to test such model of care and to better understand the epidemiology and treatment of diseases and conditions associated with DS in adulthood.

## Conflict of Interest Statement

The authors declare that the research was conducted in the absence of any commercial or financial relationships that could be construed as a potential conflict of interest.
